# The effect of different proximities to failure on arterial stiffness following resistance training protocols matched for volume‐load

**DOI:** 10.14814/phy2.70196

**Published:** 2025-04-11

**Authors:** Eleftherios Karanasios, Scott Hannah, Helen Ryan‐Stewart, James Faulkner

**Affiliations:** ^1^ School of Sport, Health and Community, Faculty of Health & Wellbeing University of Winchester Winchester UK; ^2^ School of Health and Sport Science, Faculty of Education, Humanities and Health Science Eastern Institute of Technology Napier New Zealand

**Keywords:** arterial stiffness, proximity to failure, pulse wave velocity, resistance training

## Abstract

This study compared acute changes in measures of arterial stiffness (AS) between two resistance training (RT) protocols that were load, volume and rest matched, but differed in intensity of effort. Eleven healthy adults (36.4 ± 6.8 years) performed a RT protocol with high intensity of effort (HE) and a RT protocol with low intensity of effort (LE). The HE protocol consisted of 3 sets of 12 repetitions, while the LE comprised of 6 sets of 6 repetitions. Loading intensity, volume load, and total rest duration were equivalent between the RT sessions. Pulse wave velocity, augmentation index values collected at baseline, immediately post and 15 min post‐exercise. HE elicited significantly greater increases in carotid‐femoral pulse wave velocity (6.4 ± 0.3 to 7.3 ± 0.5 m/s) when compared to LE (6.6 ± 0.3 to 6.7 ± 0.3 m/s) (*p* < 0.05). Both HE and LE induced significant increases in augmentation index (13 ± 5.6 to 28.1 ± 9.3%) post exercise (all *p* < 0.05). These findings demonstrate that RT with a lower intensity of effort attenuate increases in measures of arterial stiffness compared to a RT scheme at higher intensity of effort when volume load and total rest are equalized.

## 1 INTRODUCTION

It is well established that resistance training (RT) promotes neural and structural adaptations that synergistically increase muscular strength and hypertrophy (Suchomel et al., 2018). In addition to neuromuscular adaptations, a growing body of evidence indicates a plethora of health benefits resulting from regular participation in RT, including reductions in blood pressure, improved endothelial function, and increased quality of life (El‐Kotob et al., 2020; MacDonald et al., 2016; Silva et al., 2021). Furthermore, muscular strength, a primary adaptation to RT, is associated with lower levels of arterial stiffness (AS) (Fahs et al., 2018) and reduced mortality and cardiovascular risk in both healthy (García‐Hermoso et al., 2018) and hypertensive individuals (Artero et al., 2011). Yet, the effects of RT on the vascular system are not fully understood. Acute RT may result in a transient increase in central AS, an independent predictor of cardiovascular disease (CVD) and all‐cause mortality (Vlachopoulos et al., 2010). Measurement of carotid‐femoral pulse wave velocity (PWV) is widely accepted as the gold standard method of determining AS and has a strong predictive value for the occurrence of CVD (Kim & Kim, 2019), while the evaluation of wave reflection through the augmentation index (AIx) has been proposed as a diagnostically valuable indicator of future cardiovascular event risk especially in younger individuals (McEniery et al., 2005).

The magnitude of physiological responses induced by RT is dependent upon the acute training variables associated with RT namely, volume, loading intensity, rest duration, and repetition velocity among others (Ratamess et al., 2009). An important variable within RT, which has recently received considerable attention (Refalo et al., 2023) and is frequently the subject of debate, is that of proximity to momentary failure [i.e., the set termination end‐point where trainees fail to execute another repetition despite attempting to (Fisher et al., 2011)]. Proximity to momentary failure is indicative of the effort exerted during RT (Steele et al., 2017) and denotes the number of repetitions remaining in a given set prior to failure (Refalo et al., 2023). Proximity to failure during RT represents the intensity of effort of a given RT session, and appears to be a key factor of both neural and structural adaptations (Schoenfeld et al., 2021), as well as a main variable determining cardiovascular and metabolic responses to RT (Gjovaag et al., 2016; Gorostiaga et al., 2012). In this regard, it has been recently suggested that intensity of effort may represent an independent loading parameter potentially affecting arterial responses to acute RT (Karanasios et al., 2023).

Exaggerated peaks in systolic blood pressure (BP) and increased sympathetic outflow have been identified as potential contributors to increased AS (Figueroa et al., 2019). Interestingly, research examining physiological responses to RT among different set end points has shown that short set configurations (i.e., cluster sets), where the set is terminated a number of repetitions short of failure, attenuate acute elevations in BP and induce a lower reduction in parasympathetic modulation than longer sets performed in a closer proximity to failure (Paulo et al., 2019; Rua‐Alonso et al., 2020), even when the volume‐load and the rest duration are similar between the set configurations. For instance, Paulo et al. (2019) reported significantly lower BP values following a RT session consisting of 3 sets of 15 repetitions compared to a RT protocol of 5 sets of 9 repetitions when performed with a 20RM loading intensity. Similarly, Rua‐Alonso et al. (2020) observed that the set configuration performed closer to failure (i.e., 4 sets of 10 repetitions at a 15RM loading intensity) induced a significantly greater reduction of cardiac parasympathetic modulation than a short set configuration (i.e., 8 sets of 5 repetitions at a 15RM loading intensity).

Yet, there is a shortage of studies examining the effects of different proximities to failure on AS. To the best of our knowledge, only Rodríguez‐Pérez et al. (2020) has investigated the influence of effort on AS. The authors reported a meaningful lower (Cohen's *d* = 0.20) carotid‐femoral PWV (cfPWV) elevation for the low‐effort group compared to the high‐effort group (Rodríguez‐Pérez et al., 2020). Nonetheless, the overall volume load was not equated between the experimental conditions. For instance, the number of repetitions performed in the high‐effort group was approximately twice that of the low‐effort group at the same loading intensity (75% 1RM). Research has conclusively demonstrated that training volume is a major determinant of hypertrophic (Schoenfeld et al., 2017), cardiovascular (Figueiredo et al., 2015), and metabolic responses to RT (Strasser et al., 2010). Thus, it remains unclear whether such findings can be attributed exclusively to the different intensity of effort exerted by the aforementioned study's participants, and whether these responses persist when the volume load is equated between conditions. Considering that the effects of RT on AS and central hemodynamics are not yet fully understood, it is crucial to determine whether the acute fluctuations in arterial stiffness can also be influenced by the intensity of effort during RT.

Therefore, the purpose of this study was to investigate the influence of effort on AS and wave reflection measures by comparing a RT scheme performed to volitional failure (i.e., with higher intensity of effort) against a RT protocol performed not to failure (i.e., with lower intensity of effort) with the same loading intensity, volume‐load, and total rest duration. It was hypothesized that high intensity of effort condition would induce greater increases in AS and wave reflection indices than the low intensity of effort condition.

## 2 METHODS

### 2.1 Participants

Eleven young healthy participants (5 males, 6 females) volunteered to take part in this study. Participants were classified as recreationally active who had been participating in some form of resistance training at least once a week for the last 6 months. All participants were in a good state of health, non‐hypertensive and free of any cardiovascular, musculoskeletal, and metabolic disease. Participants were asked to refrain from strenuous physical activity for 24 h and large meals and caffeine consumption for at least 4 h prior to testing (Townsend et al., 2015). All female participants voluntarily reported that they were tested during the follicular phase of their menstrual cycle, although previous research indicates minimal influence of different menstrual cycles on AS measured by cfPWV (Priest et al., 2018). Participants also reported that they were not taking any prescribed medications known to affect vascular function. All participants signed an institutionally approved informed consent form before data collection, and the study was approved by the Faculty of Health and Wellbeing Research Ethics Committee at the University of Winchester.

### 2.2 Sample size calculation

Sample size was calculated using G*Power 3.1 (version 3.1.9.7, Heine University, Düsseldorf, Germany). Considering the paucity of research examining changes in PWV between RT protocols with different intensity of effort under volume‐equated conditions using a repeated measures design, the effect size was estimated based upon a between‐group (e.g., heavier vs. lighter load) partial eta squared ($\eta_{p}^{2}$) effect size of 0.114 reported for acute cfPWV in a previous study (Nitzsche et al., 2016). Thus, for a repeated measures within factors design, with an effect size *F* = 0.35, a significance level of 0.05, a power of 0.80, and a correlation among repeated measurements of 0.70 (Meyer et al., 2016), a sample size of 11 participants was found to be sufficient to show a difference between the HE and LE conditions while accounting for a 10% dropout.

### 2.3 Study design

Participants took part in a crossover design and reported to the research facility on three separate occasions. The first visit consisted of anthropometric measurements (i.e., height and body mass), a familiarization session in which participants were introduced to the experimental procedures and RM testing to determine individual 12RM training loads for the deadlift and bench press exercise. In the second visit, participants performed the HE RT protocol using 3 sets of 12 repetitions with 120 s rest between sets and exercises. In the third visit, participants performed the LE RT scheme using 6 sets × 6 repetitions with 45 s rest between sets and 150 s rest between exercises.

## 3 RM TESTING PROTOCOL AND EXPERIMENTAL SESSIONS

### 3.1 RM testing protocol

The maximum amount of weight lifted for 12 repetitions with proper form in the deadlift and bench press exercise was recorded as the participant's 12RM. This was determined following the guidelines proposed by the National Strength and Conditioning Association (Haff & Triplett, 2015). All RM loads were determined in no more than five attempts with rest periods of 3 min between the trials. The final weight lifted successfully for 12 repetitions for both exercises was recorded as the 12RM.

### 3.2 Experimental sessions

Two resistance training sessions comprised the experimental protocol, one for each condition investigated (i.e., HE vs. LE). The HE protocol consisted of 3 sets to volitional failure with the 12RM load and with 120 s of rest between sets and exercises (i.e., 600 s of total resting time, not including the rest interval upon termination of the last set since measurements were conducted immediately post exercise). The LE protocol consisted of the same number of repetitions and loading intensity (i.e., 12RM) completed in the HE session, and the same total resting time (i.e., 600 s) distributed between sets and exercises (i.e., 45 s between sets and 150 s between exercises). Thus, the volume‐load (sets × repetitions × load lifted), the total rest duration, and the loading intensity (i.e., 12RM) were consistent between the experimental conditions. Participants were guided by the researcher to adopt a repetition tempo of approximately 1 s concentric, 2 s eccentric, and 1 s isometric actions.

Due to the volume‐load matched design of the present study, the order of the exercise sessions could not be randomized and the LE had to follow the HE exercise session. For instance, if a participant failed to perform the expected number of repetitions (i.e., 12) in a given set, the number of completed repetitions was recorded and adjusted accordingly to the LE condition. For example, if a participant performed 12 repetitions in the first 2 sets but 10 repetitions in the last set during the HE protocol (i.e., 34 repetitions), the same participant then performed 6 repetitions in the first 5 sets and 4 repetitions in the last set during the LE protocol (i.e., 34 repetitions).

### 3.3 Hemodynamic measurements

Hemodynamic measurements were collected three times during the experimental procedure: before training (baseline), immediately post (Post), and 15 min post training (15Post). All measurements were collected in a supine position. The first hemodynamic data collection was conducted after 10 min of rest in a supine position (Townsend et al., 2015). Following that, participants completed the warm‐up routine as described above, and then completed their assigned RT protocol. Participants returned to and remained in the supine position immediately following the acute RT protocol while data collection equipment was applied. Hemodynamic measurements were conducted within 1 min (post) and 15 min (15Post) following the acute exercise session.

All hemodynamic measurements were performed using a validated oscillometric cuff‐based device (Vicorder, Skidmore medical, Bristol, UK) that calculates PWV by simultaneously recording the upstroke of the femoral and carotid pulsations (Hickson et al., 2009). Two inflatable cuffs were used to measure carotid‐femoral pulse wave velocity (cfPWV), one placed around the neck over the carotid artery and the other around the thigh over the femoral artery. CfPWV was calculated by dividing the pulse wave travel distance by the pulse transit time between the two recording sites. Transit time was determined by the software using an in‐built cross‐correlation algorithm, and travel distance length was defined as the distance from the suprasternal notch to the mid upper thigh cuff, as indicated by the manufacturer. AIx is an indicator of AS which reflects the augmentation of systolic blood pressure by reflection of the peripheral pulse wave. AIx was calculated as the ratio between the augmentation pressure (AP) and the central pulse pressure (Cpp) and was expressed as a percentage (AIx = AP/Cpp × 100). AIx was normalized to 75 bpm (AIx@75) to reduce its reliance on HR, as previously suggested (Wilkinson et al., 2000). Measurements of SEVR, an index representing myocardial perfusion, which is calculated as the ratio between diastolic pressure time index (DPTI) and systolic pressure time index (SPTI) (Tsiachris et al., 2012), and of central and peripheral blood pressures were performed using a cuff placed around the upper arm over the brachial artery and assessed using device‐specific pulse wave analysis (Baier et al., 2018).

### 3.4 Statistical analysis

All descriptive data are expressed as means ± standard deviation. Normality of distribution was assessed by the Shapiro–Wilk test. A two‐way (Condition × Time) repeated measured analysis of variance (ANOVA) was conducted to evaluate the Condition by Time interaction, and independent Condition and Time main effects for: cfPWV; AIx; AIx@75; central systolic blood pressure (CsBP); central diastolic blood pressure (CdBP); peripheral systolic blood pressure (PsBP); peripheral diastolic blood pressure (PdBP); augmentation pressure (AP); mean arterial pressure (MAP); and SEVR. Violations of sphericity were adjusted using Greenhouse–Geisser. Post‐hoc pairwise comparisons with a Bonferroni correction were conducted when Condition by Time interactions were detected. Analysis of the effect size was conducted using the partial Eta squared ($\eta_{p}^{2}$). The magnitude of effect size was interpreted as trivial (<0.01), small (0.01–0.06), moderate (>0.06–0.14), and large (>0.14) (Richardson, 2011). Significance was set at *p* < 0.05. Data were analyzed using SPSS version 28 statistical software (SPSS Inc., Chicago, IL, USA). A linear mixed‐effects model (Jamovi, version 2.3.28) was also utilized to assess the effect of the intervention on cfPWV (primary outcome), controlling for mean arterial pressure (MAP) as a covariate (Townsend et al., 2015). Random intercepts were included for each subject to account for repeated measurements over time. Fixed effects in the model included Condition, Time, and the interaction between Condition and Time. MAP was included as a covariate to adjust for its influence on cfPWV. Residual plots were examined to verify model assumptions of normality and homoscedasticity.

## 4 RESULTS

Characteristics of the study participants are displayed in Table 1.

**TABLE 1:** Participant characteristics.

Age (y)	36.4 ± 6.8
Weight (kg)	67.3 ± 12.0
Height (cm)	172.8 ± 7.8
12RM dead lift (kg)	56.6 ± 15.0
12RM bench press (kg)	40.6 ± 10.9
Volume (Repetitions)	70.4 ± 0.9
Volume load (kg)	3419.6 ± 884.8

*Note*: Volume load is calculated as: sets × repetitions × load. Data presented are mean ± SD.

Abbreviations: BP, bench press; DL, deadlift.

There were no significant differences between the experimental conditions at baseline (BL) for any of the variables analyzed.

**FIGURE 1:**
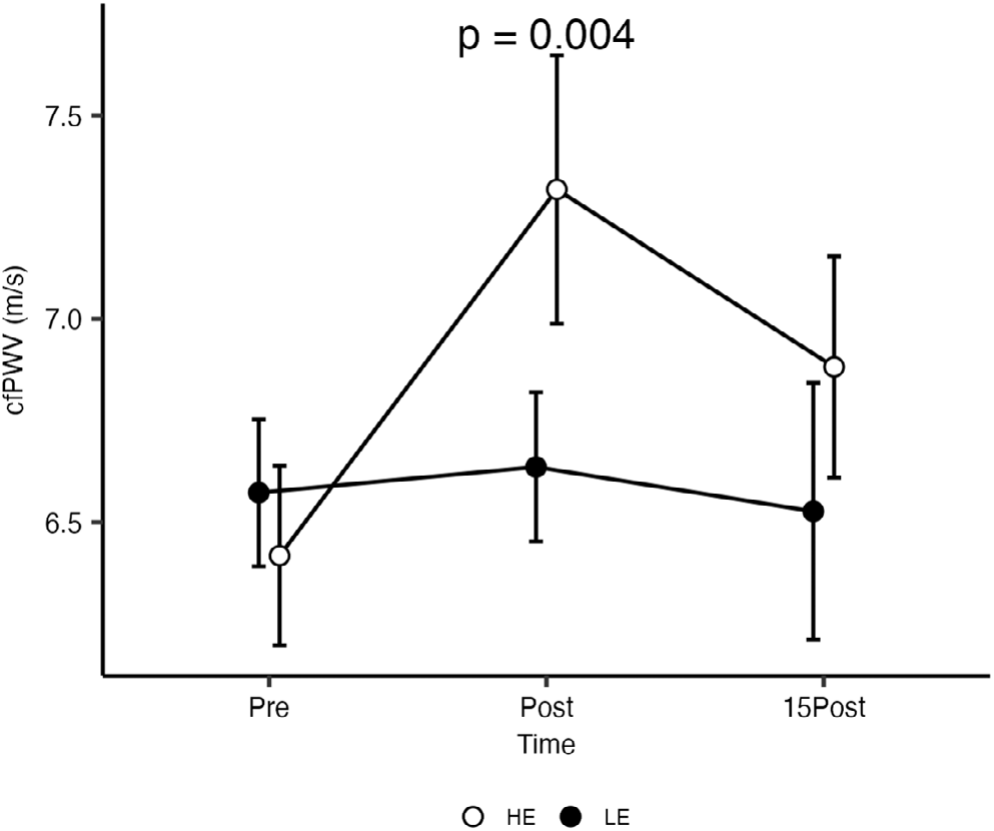
Changes in cfPWV measured at rest and following the RT protocols. CfPWV, carotid‐femoral pulse wave velocity; HE, high effort; LE, low effort; Interaction effect at the post exercise timepoint (*p* = 0.004). Data are presented as means and confidence intervals 95%.

Hemodynamic data are shown in Table 2. A significant Condition by Time interaction was observed for cfPWV ($\eta_{p}^{2}=0.64$, *p* < 0.05) with a larger increase in cfPWV from BL to Post observed in HE compared to LE (Figure 1). Further, cfPWV was significantly greater than BL at both post and 15Post but significantly decreased from Post to 15Post for the HE condition. For the LE no significant differences in cfPWV from BL were observed. MAP had no significant main effect on cfPWV (*p* = 0.06), therefore a similar statistical Condition by Time interaction was observed for cfPWV when controlling for MAP (*p* < 0.001). There were no significant Condition by Time interactions for all other variables (Table 2).

**TABLE 2:** Hemodynamic and cardiovascular variables at rest and during recovery from an acute HE and LE resistance training protocol.

	Time point			Interaction effect	
	Baseline	Post	15Post	*p* Value	Effect size (*η* ^2^_p_)
cSBP (mmHg)^c^					
HE	111.4 ± 11.0	119.9 ± 10.2	111.3 ± 14	0.268	0.12
LE	113.8 ± 11.1	117.3 ± 12.0	113.3 ± 11.2		
Total	112 ± 10.2	118.5 ± 8.5	112.2 ± 11.9		
cDBP (mmHg)^b^, ^c^					
HE	60.5 ± 8.5	47.8 ± 6.6	50.2 ± 11.0	0.157	0.16
LE	61.4 ± 8.6	54.3 ± 10.4	58.5 ± 11.9		
Total	61 ± 8	51 ± 7	54.4 ± 9		
cfPWV (m/s)^a^					
HE	6.4 ± 0.3	7.3 ± 0.5	6.9 ± 0.4	<0.001	0.64
LE	6.6 ± 0.3	6.7 ± 0.3	6.5 ± 0.5		
Total	6.4 ± 2.3	6.9 ± 2.3	6.7 ± 0.3		
cfPWV‐adjusted (m/s)^a^					
HE	6.4 ± 0.3	7.3 ± 0.3	6.8 ± 0.3	<0.001	
LE	6.6 ± 0.3	6.5 ± 0.3	6.5 ± 0.3		
Total	6.5 ± 0.3	7 ± 0.3	6.7 ± 0.3		
AIx (%)^c^					
HE	12.5 ± 5.4	32.5 ± 10.7	17.2 ± 6.2	0.078	0.25
LE	13.7 ± 8.2	23.9 ± 12.4	14.9 ± 8.1		
Total	13 ± 5.6	28.1 ± 9.3	16 ± 5.3		
AIx75@ (%)					
HE	−5.1 ± 3.3	0.6 ± 7.7	−2.0 ± 6.1	0.137	0.18
LE	−4.7 ± 3.8	−2.7 ± 6.1	−3.4 ± 3.4		
Total	−4.9 ± 3	−1 ± 5.6	−2.6 ± 4.3		
AP (mmHg)^c^					
HE	6.5 ± 3.2	23.5 ± 9.6	11 ± 7.3	0.054	0.25
LE	7.2 ± 4.6	15.8 ± 9.0	8.9 ± 5.4		
Total	6.8 ± 3.3	19.6 ± 7.6	9.9 ± 4.6		
pSBP (mmHg)					
HE	118.4 ± 8.8	123.2 ± 10.1	116.4 ± 13	0.241	0.13
LE	120.4 ± 10.1	121.2 ± 9.7	119.0 ± 9.6		
Total	119.3 ± 8.3	122.1 ± 7.6	117.6 ± 10.3		
pDBP (mmHg)^c^					
HE	60.5 ± 8.5	47.8 ± 6.6	50.2 ± 11	0.192	0.15
LE	61.8 ± 9.0	54.5 ± 10.9	58.5 ± 11.9		
Total	61.1 ± 8	51.1 ± 7	54.3 ± 9		
HR (bpm)					
HE	64.1 ± 6.8	75.5 ± 16.0	70.5 ± 12.6	0.147	0.17
LE	64.9 ± 7.9	68.8 ± 12.6	67.6 ± 7.1		
Total	64.5 ± 6.3	72.1 ± 11.9	69 ± 6		
MAP (mmHg)					
HE	83 ± 10.8	82.4 ± 8.0	79.6 ± 10.3	0.712	0.03
LE	84.8 ± 10.6	83.8 ± 9.6	83.5 ± 11.0		
Total	83.9 ± 9.9	83 ± 7.3	81.5 ± 9.3		
SEVR (%)^c^					
HE	160.6 ± 18.8	128.5 ± 26.9	131.1 ± 36.2	0.227	0.13
LE	156.9 ± 26.4	141.8 ± 30.0	142.5 ± 34.1		
Total	158.7 ± 20.9	135.1 ± 25.5	136.7 ± 32.2		

*Note*: Data are displayed as means ± SD.

Abbreviations: AIx, augmentation index; Aix@75, augmentation index normalized at 75bmp; AP, augmentation pressure; cDBP, central diastolic blood pressure; cfPWV, carotid‐femoral pulse wave velocity; cfPWV‐adjusted, cfPWV adjusted for mean arterial pressure; cSBP, central systolic blood pressure; HE, high effort; HR, heart rate; LE, low effort; MAP, mean arterial pressure; pDBP, peripheral diastolic blood pressure; pSBP, peripheral systolic blood pressure; SEVR, subendocardial viability ratio; Total, means of main effect of time.

^a^ Interaction main effect (*p* < 0.05).

^b^ Condition main effect (*p* < 0.05).

^c^ Time main effect (*p* < 0.05).

A Condition main effect was evident for cDBP ($\eta_{p}^{2}=0.36$, *p* < 0.05), with lower values observed for HE (47.8 ± 6.6 mmHg at Post and 50.2 ± 11 mmHg at 15Post) compared to LE (54.3 ± 10.4 mmHg at Post and 58.5 ± 11.9 mmHg at 15Post).

Main effects for Time were detected for AIx ($\eta_{p}^{2}=0.78$), AP ($\eta_{p}^{2}=0.76$), CsBP ($\eta_{p}^{2}=0.41$), CdBP ($\eta_{p}^{2}=0.48$), PdBP ($\eta_{p}^{2}=0.49$), and SEVR ($\eta_{p}^{2}=0.36$) (all *p* < 0.05). AIx and AP significantly increased from BL to Post and 15Post, and then significantly decreased from Post to 15Post. Increases in AIx from resting values were higher in HE than in LE condition by 26% at Post (Cohen's *d* = 0.62) and 13% at 15Post (Cohen's *d* = 0.24), respectively. For CsBP significant increases from BL were observed at Post. CdBP and PdBP significantly decreased at Post and 15Post. Regarding SEVR, significant decreases were observed at Post.

## 5 DISCUSSION

This study compared acute changes in indices of AS and central hemodynamics, between two volume and rest equivalent RT protocols with different intensity of effort. Results of the present study indicate that RT protocols performed to volitional failure induce a more pronounced hemodynamic response in comparison to RT protocols that do not elicit failure, even when volume and rest duration are equivalent. Supporting our hypothesis, changes in AS measured by cfPWV were significantly higher immediately after exercise in the HE in comparison to the LE condition. Furthermore, both RT protocols induced significant increases in AIx and CsBP and significant decreases in CdBP and SEVR, with residual effects persisting up to 15 min into recovery. Of note, this is the first study to assess AS responses to acute RT performed at different intensities of effort on a volume‐equated basis and given the paucity of similar research comparison with previous studies is limited. Results presented herein indicate that intensity of effort can significantly affect AS responses to RT independent of load, volume, and rest duration.

As proximity to failure nears with in a set during successive repetitions, metaboreflex and central command activation induce autonomic adjustments, promoting increases in cardiac output and systemic vascular resistance that collectively concur in raising BP. In the present study, CsBP significantly increased (7.7% in the HE vs. 3% in the LE) immediately post‐exercise. It has been postulated that intermittent increases in BP may shift the vessel's load‐bearing properties from the more elastic elastin to stiffer collagen fibers, thus partially explaining increases in AS (O'Rourke & Nichols, 2005). Further, it is known that central command is closely related to the intensity of effort and the fatigue experienced during RT while activation of the metaboreflex is mainly related to metabolic demands of the exercising musculature (De Morree et al., 2012; Teixeira et al., 2019). That said, research has shown that sets performed closer to failure induce more pronounced autonomic responses (i.e., increased sympathetic activation and vagal inhibition) (Rua‐Alonso et al., 2020), higher metabolite accumulation (Gorostiaga et al., 2014) and greater increases in BP (Paulo et al., 2019) compared to shorter set configurations, even when training volume is equalized. Collectively these findings indicate that RT protocols performed with a high intensity of effort promote a more distinct hemodynamic response compared to RT designs with lower intensities of effort and may partially explain findings of the present study. In addition, since is more effortful to lift the same load and terminate the set at the point of volitional failure than terminate the set six repetitions away from failure, this may have caused participants of the present study to perform the Valsalva maneuver which can be reflexively adopted as a stabilizing action when higher efforts are required (Hackett & Chow, 2013). Considering that the Valsalva maneuver can independently increase AS, via direct transmission of intra‐thoracic and intra‐abdominal pressures to the aorta (Heffernan et al., 2007), this may explain the significant differences in cfPWV observed herein.

Results of the present study are partially in line with a previous study that compared hemodynamic responses to RT at different proximities to failure (Rodríguez‐Pérez et al., 2020). Rodríguez‐Pérez et al. (2020) reported that cfPWV was higher in the high‐effort group (i.e., trainees performed repetitions to volitional failure) than the low‐effort group (i.e., trainees performed half the number of the maximum possible repetitions) immediately after a RT protocol consisting of 3 sets of bench press and squats at 75% 1RM. However, in the study by Rodríguez‐Pérez et al. (2020) training volume in the high‐effort group was twice that of the low‐effort group. Research indicates that volume is a crucial factor and may have a greater impact on physiological adaptations to RT than other RT variables. Indeed, recent evidence suggests that loading intensity and training frequency might have less of an impact on inducing improvements in BP and glycemic control when training volume is matched between experimental conditions (Correia et al., 2023; Yang et al., 2017). Thus, findings of Rodríguez‐Pérez et al. (2020) cannot be solely attributed to the different level of effort since differences in volume may have been a confounder. Further, cfPWV increased only slightly (from 5.04 to 5.26 m/s) immediately post‐training for the high‐effort group in Rodríguez‐Pérez et al. (2020) and was not statistically significant. Conversely, increases in cfPWV immediately post training (Post) presented herein, were significantly greater (from 6.4 to 7.3 m/s) than baseline values for the HE condition. Differences in the order of exercises [i.e., the last exercise performed in Rodríguez‐Pérez et al., 2020 was a lower body (squat) vs. an upper body (bench press) in the present study] may partially explain the greater magnitude of change in cfPWV observed herein, since exercises targeting the lower body tend to have less of an impact on arterial stiffening (Li et al., 2015). Future research may consider randomizing exercise order to elucidate a potential impact on AS.

Data from the present study demonstrated that cfPWV significantly increased by 14% from baseline values in the HE condition immediately post‐training. These results are in agreement with some (Kingsley et al., 2016; Parks et al., 2020) but inconsistent with others (Erb et al., 2022; Thiebaud et al., 2016). Kingsley et al. (2016) reported that cfPWV significantly increased by 9.6% in young adults 10 min after a RT protocol using free weight exercises. Conversely, Thiebaud et al. (2016) observed a nonsignificant 8.4% increase in cfPWV in young healthy adults 5 min post training. Elsewhere Parks et al. (2020) demonstrated a significant increase in cfPWV following a RT using weight machines. Whereas in a follow‐up study using the same RT protocol (i.e., 3 sets of 10 repetitions at 75% 1RM on the leg press, chest press, lat pulldown, leg extension, and leg curl exercises) and the same rest duration (i.e., 2 min between sets and exercises), Erb et al. (2022) reported no significant increases (7% in men and 6.7% in women) in cfPWV during the recovery period. Similar inconsistencies are also reported in regard to BP responses to acute RT (DeVan et al., 2005; Thiebaud et al., 2016). For instance, DeVan et al. (2005) observed a significant increase in CsBP but no change in PsBP following acute RT. In contrast, Thiebaud et al. (2016) reported opposite results with significant PsBP elevations but no alterations in CsBP. Such discrepancies highlight the complex interactions between RT variables, AS and blood pressure and further reinforce the necessity of standardization among RT variables to fully appreciate the hemodynamic response to acute RT. Regardless, data from the present study indicate that when other RT variables are held constant, intensity of effort during RT can significantly affects AS, highlighting its independent importance as previously suggested (Karanasios et al., 2023).

In the present study, AIx significantly increased post‐exercise regardless of the RT protocol. Increases in AIx from resting values were higher in HE than in LE condition by 26% at Post (Cohen's *d* = 0.62) and 13% at 15Post (Cohen's *d* = 0.24), respectively, although these differences did not reach statistical significance. Conversely, no significant changes were observed for AIx@75 throughout the recovery period. Wave reflection measures are influenced by the velocity of the pulse wave (Davies & Struthers, 2003), thus higher cfPWV values reported in the HE condition might have been responsible for the early return of the reflected wave consequently augmenting AIx. Previous studies have reported increases in AIx@75 but no changes in AIx after acute RT (Parks et al., 2020; Yoon et al., 2010), which is in contrast to the results presented herein. This discrepancy between former work and the present might be related to the influence of age on vascular responses to RT, as previous evidence suggests that young individuals demonstrate greater acute changes in AIx@75 possibly due to more compliant and vasoresponsive arterioles (Thiebaud et al., 2016). In our study the mean age of participants was 36.4 years, more than 10 years older than the Parks et al. (2020) sample (23.3 years). This may partially explain the distinct wave reflection responses. In addition, increases in AIx reported in the current study seem to be unrelated to HR since no significant changes in HR were evident. Similar findings have been elsewhere observed (Parks et al., 2020) and might indicate that increases in AIx can occur independently of changes in HR, despite previous evidence suggesting an inverse relationship between AIx and HR (Wilkinson et al., 2000).

The RT protocols adopted in the current study significantly decreased CdBP and SEVR post‐exercise. Reduction in CdBP was significantly greater in HE compared to LE. Decreases in CdBP in response to RT have elsewhere been reported (Thiebaud et al., 2016) although this finding is not universally supported (Parks et al., 2020). Similarly, decreases in SEVR reported herein are consistent with previous research reporting reductions in SEVR after acute RT (Parks et al., 2020). Given that SEVR represents the myocardial oxygen supply (DPTI) and demand (SPTI) ratio, it has been postulated that decreases in SEVR may result in transient reductions in coronary blood flow (Parks et al., 2020; Salvi & Parati, 2015). Yet, recent evidence indicates that decreases in central arterial compliance following RT (i.e., increases in AS) are negatively correlated with SPTI but not related to changes in DPTI, indicating that decreases in SEVR may mostly represent increased myocardial demand and to a lesser extent reduced coronary blood flow (Tagawa et al., 2018). Due to the scarcity of available data, further research is required to clarify potential relationships and determine whether these acute changes translate to adverse long‐term adaptations.

There are a few limitations in the present study that warrant consideration. Due to the volume‐load matched design the order of the exercise conditions could not be randomized, thus an order effect cannot be eliminated. The Valsalva maneuver was not controlled in this study even though it is known that use of the Valsalva maneuver can independently affect AS (Heffernan et al., 2007), yet participants were encouraged to breathe normally during lifting. In addition, in the current study both males and females were tested, thus sex‐related differences in arterial hemodynamics previously reported after acute endurance exercise (Doonan et al., 2013), might be a confounding factor. Nonetheless, the available literature does not indicate a sex differential response to acute RT (Erb et al., 2022; Kingsley et al., 2017). Lastly, findings of the current study are only related to the acute effects of RT on AS and cannot be used to establish arterial adaptions to chronic RT. Despite these limitations, the current study suitably controlled a set of RT variables (i.e., load, volume, and rest duration) to investigate the independent effects of effort on AS and demonstrates that without compromising training volume shorter sets with a lower intensity of effort impose a lesser demand on the cardiovascular system. In addition, the present study was structured as a within‐subjects design thus minimizing inter‐individual variability in physiological responses to RT (Grgic et al., 2022).

## 6 CONCLUSION

The current study suggests that performing RT to volitional failure promotes significantly greater acute increases in cfPWV and greater acute decreases in CdBP compared to a RT session where the set is terminated at the half point prior to failure, even when volume load and total rest duration are equated. Collectively, these data suggest that RT protocols with a higher intensity of effort impose a greater workload on the arterial and cardiovascular system in comparison to RT protocols performed with a lower intensity of effort. Findings of the present study may have important implications for exercise prescription particularly from a cardiovascular‐health standpoint. Future research should examine the potential implication of different proximities to failure (i.e., 1 repetition in reserve vs. 2 repetitions in reserve etc.), and aim to establish if the acute responses observed herein translate to long‐term arterial adaptations to RT.

## AUTHOR CONTIBUTIONS

EK was involved in conceptualization, writing/original draft preparation, and investigation/testing; EK, JF, and SH were involved in data analysis; JF, SH, and HR‐S were involved in review and editing. All authors have read and approved the final version of the manuscript and agree with the order of presentation of the authors.

## FUNDING INFORMATION

The authors received no financial support for the research authorship and/or publication of this article.

## CONFLICT OF INTEREST STATEMENT

The authors reported no conflict of interest relevant to this manuscript.

## ETHICS STATEMENT

The study was approved by the Faculty of Health and Wellbeing of the University of Winchester (HWB_REC_230811_Karanasios). Experimental procedures were conducted following the approved ethics submission document. All participants received written information explaining the procedures and purpose of the study and gave their written consent prior to data collection.

## Data Availability

The data collected and analyzed for this study are available from the corresponding author upon request.

## References

[phy270196-bib-0001] Artero, E. G. , Lee, D.‐C. , Ruiz, J. R. , Sui, X. , Ortega, F. B. , Church, T. S. , Lavie, C. J. , Castillo, M. J. , & Blair, S. N. (2011). A prospective study of muscular strength and all‐cause mortality in men with hypertension. Journal of the American College of Cardiology, 57(18), 1831–1837. 21527158 10.1016/j.jacc.2010.12.025 PMC3098120

[phy270196-bib-0002] Baier, D. , Teren, A. , Wirkner, K. , Loeffler, M. , & Scholz, M. (2018). Parameters of pulse wave velocity: Determinants and reference values assessed in the population‐based study LIFE‐adult. Clinical Research in Cardiology, 107, 1050–1061. 29766282 10.1007/s00392-018-1278-3 PMC6208658

[phy270196-bib-0003] Correia, R. R. , Veras, A. S. C. , Tebar, W. R. , Rufino, J. C. , Batista, V. R. G. , & Teixeira, G. R. (2023). Strength training for arterial hypertension treatment: A systematic review and meta‐analysis of randomized clinical trials. Scientific Reports, 13(1), 201. 36604479 10.1038/s41598-022-26583-3 PMC9814600

[phy270196-bib-0004] Davies, J. I. , & Struthers, A. D. (2003). Pulse wave analysis and pulse wave velocity: A critical review of their strengths and weaknesses. Journal of Hypertension, 21(3), 463–472. 12640232 10.1097/00004872-200303000-00004

[phy270196-bib-0005] De Morree, H. M. , Klein, C. , & Marcora, S. M. (2012). Perception of effort reflects central motor command during movement execution. Psychophysiology, 49(9), 1242–1253. 22725828 10.1111/j.1469-8986.2012.01399.x

[phy270196-bib-0006] DeVan, A. E. , Anton, M. M. , Cook, J. N. , Neidre, D. B. , Cortez‐Cooper, M. Y. , & Tanaka, H. (2005). Acute effects of resistance exercise on arterial compliance. Journal of Applied Physiology, 98(6), 2287–2291. 15718412 10.1152/japplphysiol.00002.2005

[phy270196-bib-0007] Doonan, R. J. , Mutter, A. , Egiziano, G. , Gomez, Y.‐H. , & Daskalopoulou, S. S. (2013). Differences in arterial stiffness at rest and after acute exercise between young men and women. Hypertension Research, 36(3), 226–231. 23051656 10.1038/hr.2012.158

[phy270196-bib-0008] El‐Kotob, R. , Ponzano, M. , Chaput, J. P. , Janssen, I. , Kho, M. E. , Poitras, V. J. , Ross, R. , Ross‐White, A. , Saunders, T. J. , & Giangregorio, L. M. (2020). Resistance training and health in adults: An overview of systematic reviews. Applied Physiology, Nutrition, and Metabolism, 45(10 Suppl. 2), S165–S179. 10.1139/apnm-2020-0245 33054335

[phy270196-bib-0009] Erb, E. K. , Humm, S. M. , Kearney, S. G. , Pinzone, A. G. , Kern, M. A. , & Kingsley, J. D. (2022). Sex differences in measures of wave reflection and aortic arterial stiffness in response to weight machine resistance exercise. International Journal of Exercise Science, 15(2), 1190–1201. 36619158 10.70252/WOEN6960 PMC9799230

[phy270196-bib-0010] Fahs, C. A. , Thiebaud, R. S. , Rossow, L. M. , Loenneke, J. P. , Bemben, D. A. , & Bemben, M. G. (2018). Relationships between central arterial stiffness, lean body mass, and absolute and relative strength in young and older men and women. Clinical Physiology and Functional Imaging, 38(4), 676–680. 28815984 10.1111/cpf.12467

[phy270196-bib-0011] Figueiredo, T. , Rhea, M. R. , Peterson, M. , Miranda, H. , Bentes, C. M. , dos Reis, V. M. d. R. , & Simao, R. (2015). Influence of number of sets on blood pressure and heart rate variability after a strength training session. The Journal of Strength & Conditioning Research, 29(6), 1556–1563. 25436620 10.1519/JSC.0000000000000774

[phy270196-bib-0012] Figueroa, A. , Okamoto, T. , Jaime, S. J. , & Fahs, C. A. (2019). Impact of high‐and low‐intensity resistance training on arterial stiffness and blood pressure in adults across the lifespan: A review. Pflügers Archiv ‐ European Journal of Physiology, 471, 467–478. 30426247 10.1007/s00424-018-2235-8

[phy270196-bib-0013] Fisher, J. , Steele, J. , Bruce‐Low, S. , & Smith, D. (2011). Evidence based resistance training recommendations. Medicina dello Sport, 15(3), 147–162.

[phy270196-bib-0014] García‐Hermoso, A. , Cavero‐Redondo, I. , Ramírez‐Vélez, R. , Ruiz, J. R. , Ortega, F. B. , Lee, D.‐C. , & Martínez‐Vizcaíno, V. (2018). Muscular strength as a predictor of all‐cause mortality in an apparently healthy population: A systematic review and meta‐analysis of data from approximately 2 million men and women. Archives of Physical Medicine and Rehabilitation, 99(10), 2100–2113.e2105. 29425700 10.1016/j.apmr.2018.01.008

[phy270196-bib-0015] Gjovaag, T. , Hjelmeland, A. K. , Oygard, J. B. , Vikne, H. , & Mirtaheri, P. (2016). Acute hemodynamic and cardiovascular responses following resistance exercise to voluntary exhaustion. Effects of different loadings and exercise durations. The Journal of Sports Medicine and Physical Fitness, 56(5), 616–623. 27285350

[phy270196-bib-0016] Gorostiaga, E. M. , Navarro‐Amezqueta, I. , Calbet, J. A. , Hellsten, Y. , Cusso, R. , Guerrero, M. , Granados, C. , Gonzalez‐Izal, M. , Ibanez, J. , & Izquierdo, M. (2012). Energy metabolism during repeated sets of leg press exercise leading to failure or not. PLoS One, 7(7), e40621. 22808209 10.1371/journal.pone.0040621 PMC3396634

[phy270196-bib-0017] Gorostiaga, E. M. , Navarro‐Amézqueta, I. , Calbet, J. A. , Sánchez‐Medina, L. , Cusso, R. , Guerrero, M. , Granados, C. , González‐Izal, M. , Ibáñez, J. , & Izquierdo, M. (2014). Blood ammonia and lactate as markers of muscle metabolites during leg press exercise. The Journal of Strength & Conditioning Research, 28(10), 2775–2785. 24736776 10.1519/JSC.0000000000000496

[phy270196-bib-0018] Grgic, J. , Schoenfeld, B. J. , Orazem, J. , & Sabol, F. (2022). Effects of resistance training performed to repetition failure or non‐failure on muscular strength and hypertrophy: A systematic review and meta‐analysis. Journal of Sport and Health Science, 11(2), 202–211. 33497853 10.1016/j.jshs.2021.01.007 PMC9068575

[phy270196-bib-0019] Hackett, D. A. , & Chow, C. M. (2013). The Valsalva maneuver: Its effect on intra‐abdominal pressure and safety issues during resistance exercise. Journal of Strength and Conditioning Research, 27(8), 2338–2345. 10.1519/JSC.0b013e31827de07d 23222073

[phy270196-bib-0020] Haff, G. G. , & Triplett, N. T. (2015). Essentials of strength training and conditioning 4th edition. Human kinetics.

[phy270196-bib-0021] Heffernan, K. S. , Jae, S. Y. , Edwards, D. G. , Kelly, E. E. , & Fernhall, B. (2007). Arterial stiffness following repeated Valsalva maneuvers and resistance exercise in young men. Applied Physiology, Nutrition, and Metabolism, 32(2), 257–264. 10.1139/h06-107 17486167

[phy270196-bib-0022] Hickson, S. S. , Butlin, M. , Broad, J. , Avolio, A. P. , Wilkinson, I. B. , & McEniery, C. M. (2009). Validity and repeatability of the Vicorder apparatus: A comparison with the SphygmoCor device. Hypertension Research, 32(12), 1079–1085. 19779487 10.1038/hr.2009.154

[phy270196-bib-0023] Karanasios, E. , Ryan‐Stewart, H. , & Faulkner, J. (2023). The acute effects of resistance training on arterial stiffness: A systematic review. Journal of Trainology, 12(1), 5–13.

[phy270196-bib-0024] Kim, H.‐L. , & Kim, S.‐H. (2019). Pulse wave velocity in atherosclerosis. Frontiers in Cardiovascular Medicine, 6, 41. 31024934 10.3389/fcvm.2019.00041 PMC6465321

[phy270196-bib-0025] Kingsley, J. D. , Mayo, X. , Tai, Y. L. , & Fennell, C. (2016). Arterial stiffness and autonomic modulation after free‐weight resistance exercises in resistance trained individuals. The Journal of Strength & Conditioning Research, 30(12), 3373–3380. 27253837 10.1519/JSC.0000000000001461

[phy270196-bib-0026] Kingsley, J. D. , Tai, Y. L. , Mayo, X. , Glasgow, A. , & Marshall, E. (2017). Free‐weight resistance exercise on pulse wave reflection and arterial stiffness between sexes in young, resistance‐trained adults. European Journal of Sport Science, 17(8), 1056–1064. 28671855 10.1080/17461391.2017.1342275

[phy270196-bib-0027] Li, Y. , Bopp, M. , Botta, F. , Nussbaumer, M. , Schäfer, J. , Roth, R. , Schmidt‐Trucksäss, A. , & Hanssen, H. (2015). Lower body vs. upper body resistance training and arterial stiffness in young men. International Journal of Sports Medicine, 36, 960–967. 26212244 10.1055/s-0035-1549921

[phy270196-bib-0028] MacDonald, H. V. , Johnson, B. T. , Huedo‐Medina, T. B. , Livingston, J. , Forsyth, K. C. , Kraemer, W. J. , Farinatti, P. T. , & Pescatello, L. S. (2016). Dynamic resistance training as stand‐alone antihypertensive lifestyle therapy: A meta‐analysis. Journal of the American Heart Association, 5(10), e003231. 27680663 10.1161/JAHA.116.003231 PMC5121472

[phy270196-bib-0029] McEniery, C. M. , Yasmin, n. , Hall, I. R. , Qasem, A. , Wilkinson, I. B. , Cockcroft, J. R. , & Investigators, A . (2005). Normal vascular aging: Differential effects on wave reflection and aortic pulse wave velocity: The Anglo‐Cardiff Collaborative Trial (ACCT). Journal of the American College of Cardiology, 46(9), 1753–1760. 16256881 10.1016/j.jacc.2005.07.037

[phy270196-bib-0030] Meyer, M. L. , Tanaka, H. , Palta, P. , Patel, M. D. , Camplain, R. , Couper, D. , Cheng, S. , Al Qunaibet, A. , Poon, A. K. , & Heiss, G. (2016). Repeatability of central and peripheral pulse wave velocity measures: The atherosclerosis risk in communities (ARIC) study. American Journal of Hypertension, 29(4), 470–475. 26232036 10.1093/ajh/hpv127 PMC4850900

[phy270196-bib-0031] Nitzsche, N. , Weigert, M. , Baumgärtel, L. , Auerbach, T. , Schuffenhauer, D. , Nitzsche, R. , & Schulz, H. (2016). Acute effects of different strength training protocols on arterial stiffness in healthy subjects. Group, 6, 197–202.

[phy270196-bib-0032] O'Rourke, M. F. , & Nichols, W. W. (2005). Aortic diameter, aortic stiffness, and wave reflection increase with age and isolated systolic hypertension. Hypertension, 45(4), 652–658. 15699456 10.1161/01.HYP.0000153793.84859.b8

[phy270196-bib-0033] Parks, J. C. , Marshall, E. M. , Tai, Y. L. , & Kingsley, J. D. (2020). Free‐weight versus weight machine resistance exercise on pulse wave reflection and aortic stiffness in resistance‐trained individuals. European Journal of Sport Science, 20(7), 944–952. 31662038 10.1080/17461391.2019.1685007

[phy270196-bib-0034] Paulo, A. C. , Tricoli, V. , Queiroz, A. C. , Laurentino, G. , & Forjaz, C. L. (2019). Blood pressure response during resistance training of different work‐to‐rest ratio. The Journal of Strength & Conditioning Research, 33(2), 399–407. 28658080 10.1519/JSC.0000000000002074

[phy270196-bib-0035] Priest, S. E. , Shenouda, N. , & MacDonald, M. J. (2018). Effect of sex, menstrual cycle phase, and monophasic oral contraceptive pill use on local and central arterial stiffness in young adults. American Journal of Physiology. Heart and Circulatory Physiology, 315(2), H357–H365. 29677465 10.1152/ajpheart.00039.2018 PMC6139630

[phy270196-bib-0036] Ratamess, N. A. , Alvar, B. A. , Evetoch, T. E. , Housh, T. J. , Ben Kibler, W. , Kraemer, W. J. , & Triplett, N. T. (2009). Progression models in resistance training for healthy adults. Medicine and Science in Sports and Exercise, 41(3), 687–708. 19204579 10.1249/MSS.0b013e3181915670

[phy270196-bib-0037] Refalo, M. C. , Helms, E. R. , Trexler, E. T. , Hamilton, D. L. , & Fyfe, J. J. (2023). Influence of resistance training proximity‐to‐failure on skeletal muscle hypertrophy: A systematic review with meta‐analysis. Sports Medicine, 53(3), 649–665. 36334240 10.1007/s40279-022-01784-y PMC9935748

[phy270196-bib-0038] Richardson, J. T. (2011). Eta squared and partial eta squared as measures of effect size in educational research. Educational Research Review, 6(2), 135–147.

[phy270196-bib-0039] Rodríguez‐Pérez, M. A. , Alcaraz‐Ibanez, M. , Lorente‐Camacho, D. , & Garcia‐Ramos, A. (2020). Does the level of effort during resistance training influence arterial stiffness and blood pressure in young healthy adults? Isokinetics and Exercise Science, 28(4), 375–382.

[phy270196-bib-0040] Rua‐Alonso, M. , Mayo, X. , Mota, J. , Kingsley, J. D. , & Iglesias‐Soler, E. (2020). A short set configuration attenuates the cardiac parasympathetic withdrawal after a whole‐body resistance training session. European Journal of Applied Physiology, 120(8), 1905–1919. 32583361 10.1007/s00421-020-04424-3

[phy270196-bib-0041] Salvi, P. , & Parati, G. (2015). Aortic stiffness and myocardial ischemia. Journal of Hypertension, 33(9), 1767–1771. 10.1097/hjh.0000000000000706 26244626

[phy270196-bib-0042] Schoenfeld, B. J. , Grgic, J. , Van Every, D. W. , & Plotkin, D. L. (2021). Loading recommendations for muscle strength, hypertrophy, and local endurance: A re‐examination of the repetition continuum. Sports, 9(2), 32. 33671664 10.3390/sports9020032 PMC7927075

[phy270196-bib-0043] Schoenfeld, B. J. , Ogborn, D. , & Krieger, J. W. (2017). Dose‐response relationship between weekly resistance training volume and increases in muscle mass: A systematic review and meta‐analysis. Journal of Sports Sciences, 35(11), 1073–1082. 27433992 10.1080/02640414.2016.1210197

[phy270196-bib-0044] Silva, J. K. T. , Menêses, A. L. , Parmenter, B. J. , Ritti‐Dias, R. M. , & Farah, B. Q. (2021). Effects of resistance training on endothelial function: A systematic review and meta‐analysis. Atherosclerosis, 333, 91–99. 34399984 10.1016/j.atherosclerosis.2021.07.009

[phy270196-bib-0045] Steele, J. , Fisher, J. , Giessing, J. , & Gentil, P. (2017). Clarity in reporting terminology and definitions of set endpoints in resistance training. Muscle & Nerve, 56(3), 368–374. 28044366 10.1002/mus.25557

[phy270196-bib-0046] Strasser, B. , Siebert, U. , & Schobersberger, W. (2010). Resistance training in the treatment of the metabolic syndrome: A systematic review and meta‐analysis of the effect of resistance training on metabolic clustering in patients with abnormal glucose metabolism. Sports Medicine, 40, 397–415. 20433212 10.2165/11531380-000000000-00000

[phy270196-bib-0047] Suchomel, T. J. , Nimphius, S. , Bellon, C. R. , & Stone, M. H. (2018). The importance of muscular strength: Training considerations. Sports Medicine, 48, 765–785. 29372481 10.1007/s40279-018-0862-z

[phy270196-bib-0048] Tagawa, K. , Choi, Y. , Ra, S.‐G. , Yoshikawa, T. , Kumagai, H. , & Maeda, S. (2018). Resistance training‐induced decrease in central arterial compliance is associated with decreased subendocardial viability ratio in healthy young men. Applied Physiology, Nutrition, and Metabolism, 43(5), 510–516. 10.1139/apnm-2017-0449 29253352

[phy270196-bib-0049] Teixeira, A. L. , Samora, M. , & Vianna, L. C. (2019). Muscle metaboreflex activation via postexercise ischemia as a tool for teaching cardiovascular physiology for undergraduate students. Advances in Physiology Education, 43(1), 34–41. 30540204 10.1152/advan.00174.2018

[phy270196-bib-0050] Thiebaud, R. S. , Fahs, C. A. , Rossow, L. M. , Loenneke, J. P. , Kim, D. , Mouser, J. G. , Beck, T. W. , Bemben, D. A. , Larson, R. D. , & Bemben, M. G. (2016). Effects of age on arterial stiffness and central blood pressure after an acute bout of resistance exercise. European Journal of Applied Physiology, 116, 39–48. 26275787 10.1007/s00421-015-3242-5

[phy270196-bib-0051] Townsend, R. R. , Wilkinson, I. B. , Schiffrin, E. L. , Avolio, A. P. , Chirinos, J. A. , Cockcroft, J. R. , Heffernan, K. S. , Lakatta, E. G. , McEniery, C. M. , & Mitchell, G. F. (2015). Recommendations for improving and standardizing vascular research on arterial stiffness: A scientific statement from the American Heart Association. Hypertension, 66(3), 698–722. 26160955 10.1161/HYP.0000000000000033 PMC4587661

[phy270196-bib-0052] Tsiachris, D. , Tsioufis, C. , Syrseloudis, D. , Roussos, D. , Tatsis, I. , Dimitriadis, K. , Toutouzas, K. , Tsiamis, E. , & Stefanadis, C. (2012). Subendocardial viability ratio as an index of impaired coronary flow reserve in hypertensives without significant coronary artery stenoses. Journal of Human Hypertension, 26(1), 64–70. 21228823 10.1038/jhh.2010.127

[phy270196-bib-0053] Vlachopoulos, C. , Aznaouridis, K. , & Stefanadis, C. (2010). Prediction of cardiovascular events and all‐cause mortality with arterial stiffness: A systematic review and meta‐analysis. Journal of the American College of Cardiology, 55(13), 1318–1327. 20338492 10.1016/j.jacc.2009.10.061

[phy270196-bib-0054] Wilkinson, I. B. , MacCallum, H. , Flint, L. , Cockcroft, J. R. , Newby, D. E. , & Webb, D. J. (2000). The influence of heart rate on augmentation index and central arterial pressure in humans. The Journal of Physiology, 525(Pt 1), 263–270. 10811742 10.1111/j.1469-7793.2000.t01-1-00263.x PMC2269933

[phy270196-bib-0055] Yang, P. , Swardfager, W. , Fernandes, D. , Laredo, S. , Tomlinson, G. , Oh, P. I. , & Thomas, S. (2017). Finding the optimal volume and intensity of resistance training exercise for type 2 diabetes: The FORTE study, a randomized trial. Diabetes Research and Clinical Practice, 130, 98–107. 28601003 10.1016/j.diabres.2017.05.019

[phy270196-bib-0056] Yoon, E. S. , Jung, S. J. , Cheun, S. K. , Oh, Y. S. , Kim, S. H. , & Jae, S. Y. (2010). Effects of acute resistance exercise on arterial stiffness in young men. Korean Circulation Journal, 40(1), 16–22. 20111648 10.4070/kcj.2010.40.1.16 PMC2812793

